# Supplementary material for: Uncertainty-aware traction force microscopy
Source: PLoS Comput Biol. 2025 Jun 12;21(6):e1013079. doi: 10.1371/journal.pcbi.1013079 (PMC12251289; doi:10.1371/journal.pcbi.1013079)
Supplement: S1 Text — Supplementary information. (PDF) [file pcbi.1013079.s001.pdf]

S1 Text

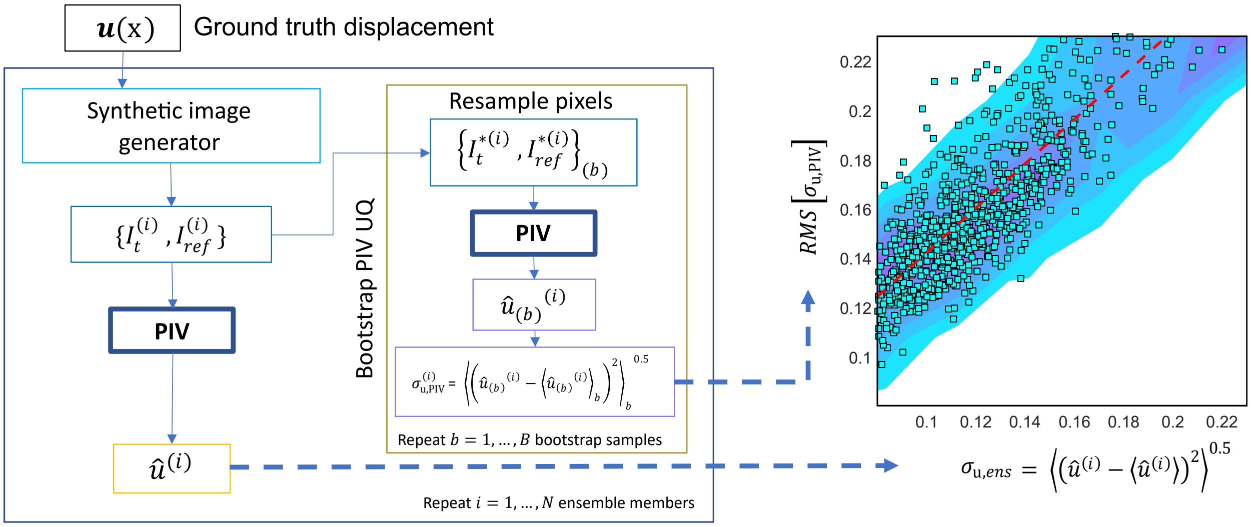

Fig A. PIV-UQ synthetic validation workflow. A fixed, high-resolution ground truth displacement $\mathbf{u}(\mathbf{x})$ is generated. One realization of synthetic images corresponding to the ground truth displacement is obtained as follows : Synthetic beads are simulated by randomly placing Gaussian beads of prescribed density with a maximum brightness of 1000 a.u. ($\boldsymbol{I}_{ref}$). The centroid of the Gaussian beads are shifted according to $\mathbf{u}(\mathbf{x}_{bead})$ to generate $\boldsymbol{I}_t$. PIV is performed to generate a displacement vector for each interrogation window, $\hat{\mathbf{u}}$. This process is repeated N times to obtain an ensemble of synthetic image and $\hat{\mathbf{u}}$ realizations that provide the true ensemble standard deviation, $\sigma_{u,ens}$. For each of the ith ensemble image set $\{\boldsymbol{I}_t^{(i)}, \boldsymbol{I}_{ref}^{(i)}\}$, PIV-UQ method is performed by bootstrapping n_B times generating an $\sigma_{u,\mathrm{PIV}}^{(i)}$ for each ensemble realization i. The root mean square (RMS) of $\sigma_{u,\mathrm{PIV}}$ is reported over N ensemble realizations for each interrogation window. The scatter plot depicts the point-wise uncertainty of true ensemble realizations with that of PIV-UQ estimate for a shear displacement field ($\mathbf{u}(\mathbf{x}) = \mathbf{u_2}$ (13)) showing the ability of PIV-UQ to predict spatial dependence of PIV uncertainty in non-uniform displacement. Overlaid heatmap shows the conditional distribution of $\sigma_{u,\mathrm{PIV}}$ given $\sigma_{u,ens}$. Black dotted line represents the best linear fit to the point cloud, showing correlation with a non-zero bias representing the floor error level.

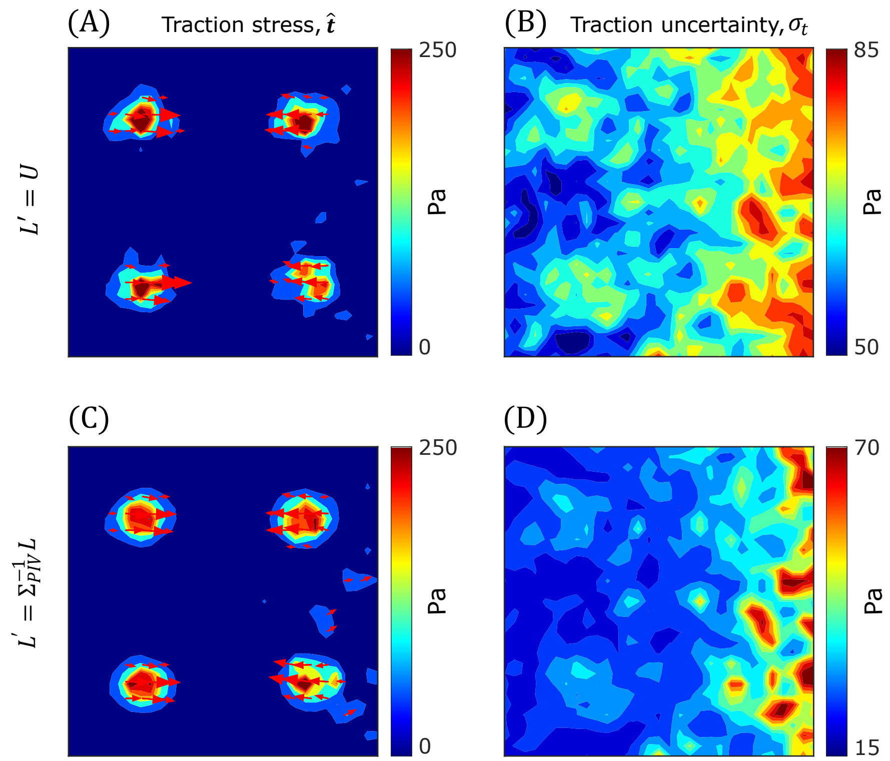

Fig B. TFM-UQ uncertainty propagation is dependent on the choice of prior. TFM-UQ is applied to synthetic simulation described in Fig 6 with the choice of two different precision matrices : identity $\mathbf{L}' = \mathbf{U}$ (A-B) and heteroskedastic prior matrix $\mathbf{L}' = \mathbf{\Sigma}_{\mathrm{PIV}}^{-1}\mathbf{L}$ (C-D). Here, $\mathbf{L}$ is the discrete Laplacian operator with second-order finite differences. The prior is of the form $p(\boldsymbol{t}|\alpha) \propto \exp\left[\frac{\alpha}{2}\boldsymbol{t}^T\mathbf{L}'\boldsymbol{t}\right]$. (A,C) Mean marginal posterior traction stress magnitude $\hat{\mathbf{t}}$ and (B,D) Marginal posterior traction uncertainty (σ_t). The spatial range of traction stress uncertainty is increased with the heteroskedastic prior (D) as opposed to $\mathbf{\Sigma}_{\mathrm{PIV}}^{-1}\mathbf{L}$ as compared to $\mathbf{L}$ (Fig 7). However, this comes at the cost of under-regularization or over-fitting (C), because the regularization favours the elastostatic term (input measurement) even in the region of higher measurement uncertainty $\mathbf{\Sigma}_{\mathrm{PIV}}$.

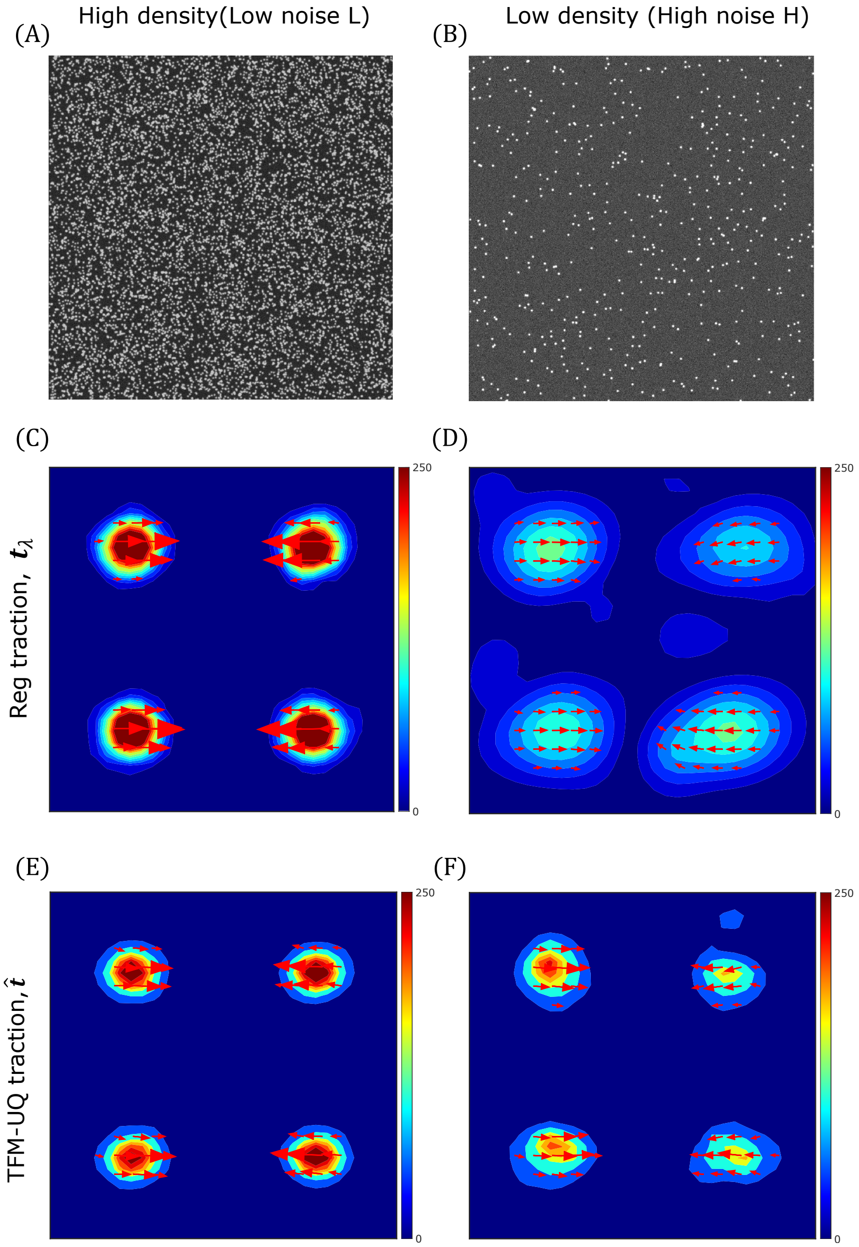

Fig C. Comparison of TFM-UQ with traditional regularizer on synthetic images of spatially uniform bead density. Two synthetic images with high (A) or low (B) bead densities are generated (corresponding to maximum an minimum densities in Fig 7 D). (C,D) Conventional TFM with a Tikhonov regularizer and Laplacian kernel $\mathbf{L}$ with L-curve criterion (λ_H and for high noise and λ_L for low noise) (E,F) TFM-UQ pipeline employing Laplacian kernel $\mathbf{L}$ is applied to synthetic traction field with ground truth illustrated in Fig 6 A.

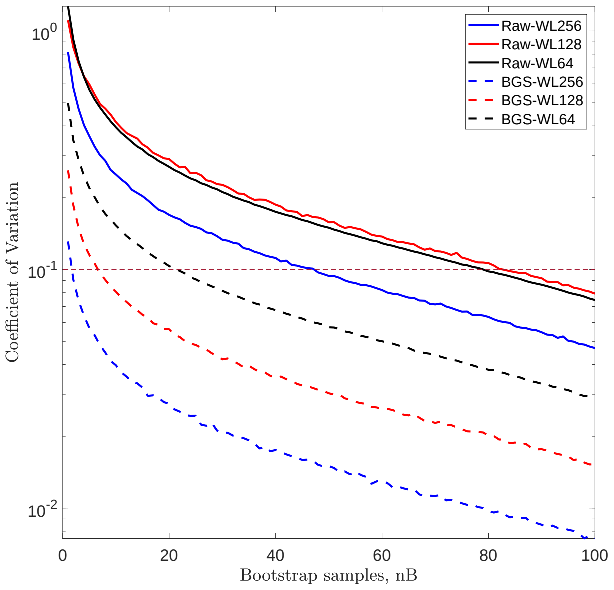

Fig D. Coefficient of variation as a metric to assess PIV-UQ convergence and the variability of $\sigma_{u,\mathbf{PIV}}$ estimate. Coefficient of variation (CoV) $= \text{Std}[\sigma_{u,\text{PIV}}]/\,\text{Mean}[\sigma_{u,\text{PIV}}]$. "Raw" images tend to need higher iterations for the error in $\sigma_{u,\text{PIV}}$ estimation to decay to 10% variation compared to "BGS" (Background subtracted) images in a resolution dependent manner.

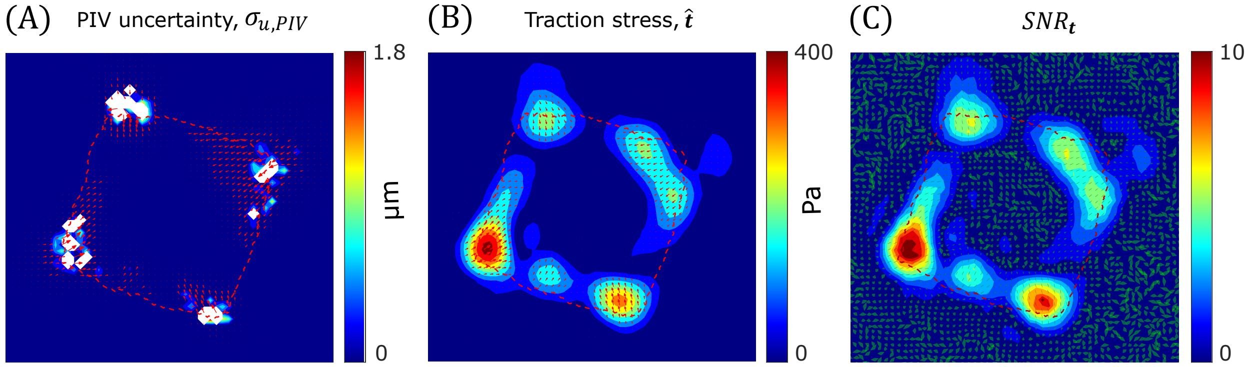

Fig E. C3H/10T1/2 fibroblast spreading on fibronectin micropatterned island, analyzed with PIV window size $\mathbf{W_L = 32}$. (A) PIV measurement, $\boldsymbol{u}_{\text{PIV}}$ (arrows) overlaid on PIV-UQ uncertainty field ($\sigma_{u,\text{PIV}}$). White region indicates bad windows that are deleted during PIV-UQ validation (B) Mean marginal posterior traction stress field ($\hat{\mathbf{t}}$) showing smoothness prior in action in the place of bad PIV windows. (C) Traction stress signal-to-noise ratio ($SNR_{\boldsymbol{t}}$). Uncertainty arrows denote the pointwise angular uncertainty corresponding to 1 circular std. dev. of marginal posterior $p(\boldsymbol{t}|\boldsymbol{u}_{\text{h}})$. Compared to WL-64 (Fig 8), a reduction in $SNR_{\boldsymbol{t}}$ was observed everywhere except the lower left traction corner of the micropatterened island (due to increase in the number of bad PIV windows in the corners). A higher traction stress magnitude is also observed only in the lower left corner where $SNR_{\boldsymbol{t}}$ is sustained from WL-64. Dashed outline indicate cell contour.

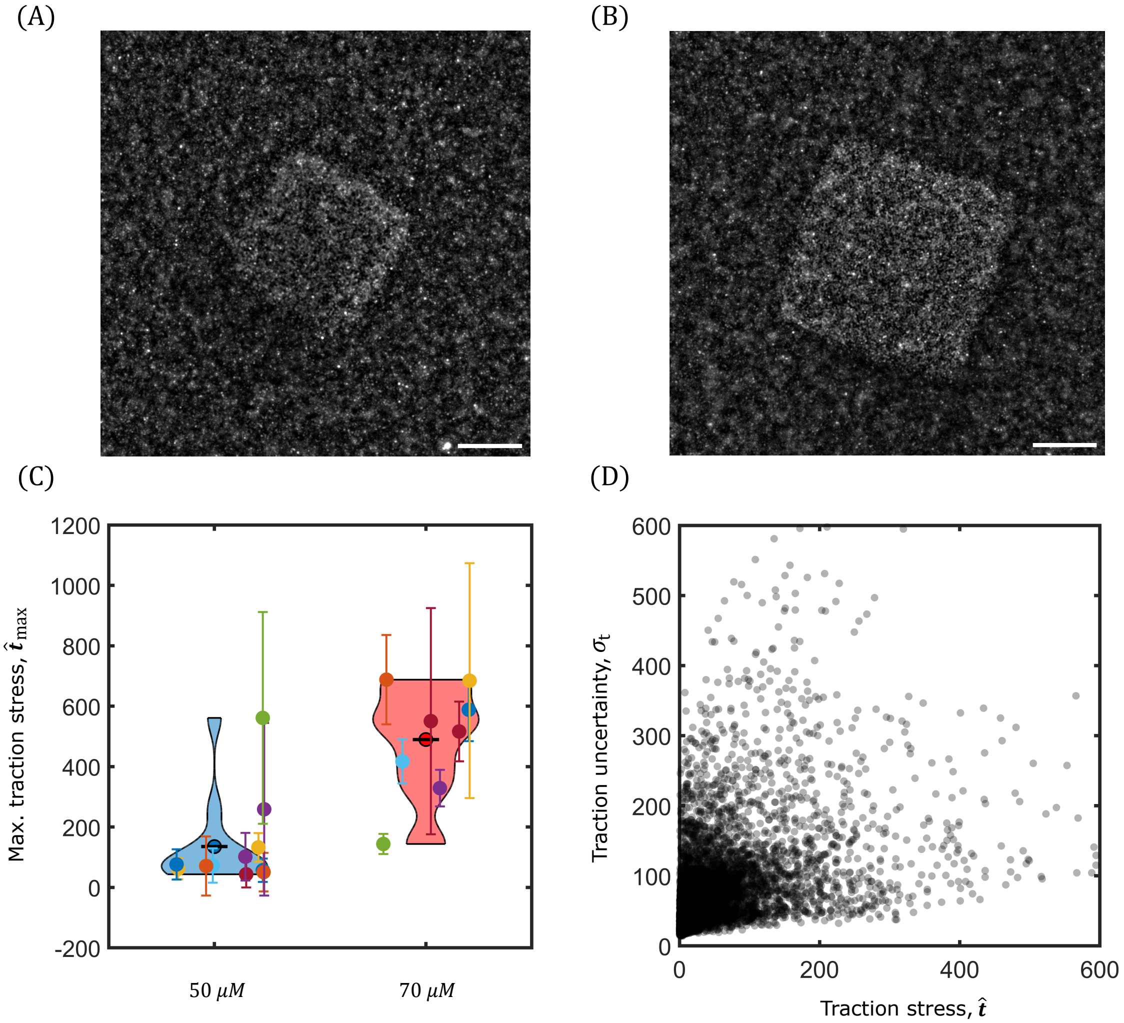

Fig F. Application of TFM-UQ to C3H/10T1/2 fibroblasts spreading on fibronectin micropatterned island of different sizes differentiates TFM-UQ measurement uncertainty from biological variability. Representative fluorescent substrate bead images showing fibronectin stamped island (square) of side 50 (A) and 75μm (B) respectively. C3H/10T1/2 fibroblasts are cultured on the micropatterened islands. (C) Distribution of max traction stress per cell $|\hat{\mathbf{t}}_{max}|$ and their respective posterior uncertainties for cells cultured on fibronectin pattern of length 50 ($n = 11$) and 75μm ($n = 8$). Violin plot visualizes the distribution of $\hat{\mathbf{t}}_{max}$ (solid points) and errorbar corresponds to individual posterior traction (TFM measurement) standard deivation. (D) Scatter plot of recovered pointwise traction stresses of all cells and the associated pointwise posterior standard deviation. Scale bar : 25 μm.
