# Supplementary material for: Uncertainty-aware traction force microscopy
Source: PLoS Comput Biol. 2025 Jun 12;21(6):e1013079. doi: 10.1371/journal.pcbi.1013079 (PMC12251289; doi:10.1371/journal.pcbi.1013079)
Supplement: Attachment — Submitted filename: TFMUQ-revision-reviewerscomments.pdf [file pcbi.1013079.s002.pdf]

Reviewers' Comments and Response to Reviewers

Reviewer #1

This is a method development manuscript on the topic of traction force microscopy (TFM), which is a method to quantify traction forces produced by cells by measuring displacements in the substrate beneath the cells. There are multiple technical challenges in TFM, with variable image quality and sensitivity to noise being major challenges. The main contribution of this manuscript is that it describes methods to handle those common challenges. The approach used here is to quantify uncertainty in the measured substrate displacements, which in turn is used to determine the optimal regularization/smoothing parameters used in the calculation of traction forces. In this approach, the regularization parameters can vary over space. Questions about the manuscript are listed below, approximately in order of importance.

Authors' response: We thank this reviewer for recognizing that our work addresses significant challenges in TFM and for the constructive feedback. We have revised our manuscript incorporating all the reviewer's suggestions, as detailed below.

1. The first, and most important, comment is about how the authors envision this method being used. The introduction (line 70 of page 4) suggests that a reason that TFM is not commonly used is that it is technically challenging: "perpetuating the view that TFM is technically involved and challenging to interpret, hindering its more widespread adoption." The implication here is that TFM is not widely used because the technical details are complex. The present manuscript presents a method for TFM that is substantially more complex than the commonly used methods. It would seem that this added complexity would further hinder adoption of TFM, unless the authors are proposing that TFM calculations become a black box, with no need for the user to understand the process. For the method described in this manuscript, the black box approach would be highly problematic, because the regularization parameters can vary over space due to changes in image quality, which in turn affects the traction forces output by the computations. If a user were unaware that the regularization varies in space depending on image quality, they could mistakenly infer changes in the traction data as being the result of biological changes rather than variation in the image quality. For this reason, a black box approach is not possible, and the users will require some understanding of the details of how the method is implemented. Thus, the notion that this method could widen the user base for TFM seems to be incorrect. With this in mind, the authors should consider who is the ideal user for this method – what expertise is needed and what type of experiment would benefit from this method? And what type of experiment could be successfully done with simpler implementations of TFM? These details should be explained in the introduction and discussion/conclusions.

   Authors' response: We appreciate these comments and suggestions. We have revised the introduction and discussion sections to address them.

   First, we have clarified that users often find TFM complex not due to the technical intricacy of its algorithms but because understanding these intricacies is necessary for parameterizing the algorithms and interpreting results. We also emphasize that even for those familiar with elastostatic inversion, choosing regularization parameters remains largely subjective in current methods.

   Second, we highlight the importance of the posterior uncertainty maps in TFM-UQ (Bayesian TFM). Unlike traditional methods, TFM-UQ allows users to obtain traction force and uncertainty maps without explicitly setting regularization parameters. These maps help non-experts assess whether traction changes stem from biological effects or image quality variations. Importantly, Bayesian TFM is not a black-box approach—it provides transparency on how the algorithm balances elastostatic noise propagation with regularization errors.

   Third, the revised discussion includes guidelines for selecting a Bayesian or traditional TFM method based on experimental parameters. While TFM-UQ offers automated image quality assessment through uncertainty quantification, it comes at a higher computational cost. We argue that TFM-UQ is especially valuable when image quality varies across pixels or requires extensive user evaluation. In contrast, traditional TFM can produce similar traction maps for high-quality images with lower computational demand. Ultimately, if runtime is not a limiting factor, we recommend TFM-UQ for its added insights from posterior uncertainty maps. This point is emphasized in the revised §3.2 with addition of new fig SI.3 according to the reviewer's comment.

2. Figure 7 shows a representative result of the method wherein the image has lower quality on the right side, compared to the left, which creates a greater uncertainty on the right side compared to left. As a result, the adaptive TFM approach described here uses a higher value of the regularization parameter on the right side compared to left. Does this result in a lower magnitude of recovered traction on the right side of the image compared to left? If yes, it would see like this causes substantial challenges in interpreting the data – here a change in tractions can result from either a change in force produced by the cell or a change in regularization parameter. In other implementations of TFM, the regularization parameter is kept constant. In those other implementations, an experienced user might be able to see anomalies in the recovered tractions due to gradients in image quality and exclude that image from the data set. Here, the variable regularization seems to obscure effects of variable image quality, which could lead the user to conclude, incorrectly, that the magnitude of cell traction varies over space. How can the user of this method resolve this challenge that in this method, the tractions output are a combination of the forces produced by the cells and the spatial variability of the regularization parameter?

   Authors' response: We appreciate this comment and have revised the manuscript to emphasize the following points:

   - We underline the significance of the posterior uncertainty maps produced by Bayesian TFM. Experienced and non-experienced users can easily interpret the joint information provided by the traction maps *and* uncertainty maps to infer where the method is applying more or less regularization. In the example pointed by the reviewer, the posterior uncertainty is significantly higher towards the right of the image, indicating that the traction stress measurement is subject to more uncertainty in that region. Therefore, we surmise that Bayesian TFM does not obscure the effects of image quality in the result. On the contrary, it reveals how the regularizer is functioning.
   - In contrast, frequentist approaches are known to mask errors by introducing global over-regularization (See [1]). Without incorporating local uncertainty into traction stress recovery, users often end up with overly smoothed traction fields (e.g., Figure 7F), creating false confidence in the results. Though less frequent, TFM users may also obtain spurious traction stress foci in high-noise regions (e.g., Figure 7E). All these errors are difficult to detect without posterior uncertainty maps like those provided by Bayesian TFM, necessitating careful visual inspection of experimental images.
   - Thus, posterior traction uncertainty maps play a key role in automating quality control. While an experienced TFM user might recognize anomalies due to image gradients, manually inspecting experimental images is time-consuming and labor-intensive. With two decades of experience using TFM for cell migration studies—analyzing thousands of cells across hundreds of time frames—and for high-throughput cardiac contractility assays in drug screening, we are well aware of the inefficiency and frustration researchers face in such tasks. Uncertainty maps provide an effective solution, allowing automation by flagging experiments where uncertainty exceeds a defined threshold. We believe this feature of Bayesian TFM benefits both experts and non-experts alike.
   - Finally, the revised manuscript briefly clarifies that Bayesian TFM regularizes traction stress fields while preserving total forces. Returning to the example in Figure 7, the spatially varying regularization not only reduces but also redistributes stresses on the right side, ensuring that the total recovered forces—represented by the surface area of the stress—remain balanced between the left and right sides. This conservation can be achieved using a Laplacian regularizer, as follows from a corollary of Gauss's theorem: the integral of a scalar field (f) is not affected by a Laplacian regularizer inside a domain (Ω) if the field's gradient (∇f) vanishes at the domain's boundary ($\partial\Omega$), i.e.,

   $$\int_\Omega (1+\lambda\nabla^2) f d\Omega = \int_\Omega \left[1+\lambda\nabla\cdot(\nabla f)\right] d\Omega = \int_\Omega f d\Omega + \int_{\partial\Omega} \nabla f\cdot\vec{n}_{\partial\Omega}\, d\partial\Omega = \int_\Omega f d\Omega.$$

   In summary, we argue that the regularizer in Bayesian TFM locally redistributes traction stresses in regions where image noise prevents precise localization, while preserving the total recovered force. Crucially, Bayesian TFM maintains resolution in other areas and provides posterior uncertainty maps, enabling users to identify where regularization is applied most strongly. In contrast, traditional regularization methods degrade traction force resolution even in regions with good image quality if distant areas suffer from poor image quality. Moreover, depending on the differential operator used, these methods may or may not conserve total forces.

3. To quantify uncertainty in the displacement field recovered by particle image velocimetry (PIV), the authors propose a bootstrapping approach, wherein pixels in a PIV window are sampled randomly with replacement, which creates a bootstrapped window that is used in the PIV code for estimating the displacement of that window. This approach seems to be problematic for two reasons. First, the spatial intensity of the pixels in the window are correlated in space, because a fluorescent particle imaged by the microscopy is typically larger than a single pixel. Doesn't the bootstrap approach assume that the samples are independent? Second, this bootstrapping approach does not seem to be a physically

meaningful means of representing noise that can occur during imaging. The boostrapping leaves some pixels blank (with values of 0), which does not occur when imaging, unless there's a dead pixel in the camera. It seems that the bootstrapping approach is not a very good choice for estimating uncertainty in the PIV data. Would it be better to take a different approach? One idea is repeating the analysis after adding noise to the PIV window, though maybe the authors can think of a better method. Alternatively, if the bootstrapping is the best approach, the authors must describe in the manuscript why the two concerns above are not relevant.

Authors' response: We appreciate this thoughtful comment. We have revised the manuscript to clarify that bootstrapping pixels is a standard mathematical method to perturb the cross-correlation functional (Fig 2A, i.e. contribution of each pixel to the displacement measurement), not to simulate any particular mechanism of imaging failure, like a dead pixel in a camera. The authors agree with the reviewer that neighboring pixels are correlated in the cross-correlation functional and are completely independent. An alternate approach is to black out blocks of contiguous pixels, requiring another parameter controlling the size of the blocks . Both of these methods as well as other bootstrapping strategies have been tested in the context of optimization-based image registration [2] with no significant differences across different bootstrapping strategies. Bootstrapping individual pixels is the most simple and parameter-free method that we have adoped. While appealing in principle, adding noise to the image can effectively simulate camera pixel noise only and introduces significant arbitrariness associated with the imaging modality, optical settings, camera, substrate composition (e.g., bead distribution), and substrate deformation. The result of these interacting factors cannot be simply replicated by adding a noise distribution to a recorded image and the model of the image generation process is not readily available.

4. The manuscript shows some comparisons to Tikhonov regularization, which is one of the common approaches to handle noise. Another common approach is to use a constraint rather than regularization. A simple and physically meaningful method for constraint is to use the displacement field as a boundary condition in a finite element model for the substrate – this constraint enforces both conservation of momentum and geometric compatibility. An advantage of this constraint-based approach is that there is no need to choose a regularization parameter. How does the author's method compare to a constrained case such as this one?

   Authors' response: We agree with the reviewer that FEM model provides an alternative formulation of the operator M for traction force reconstruction. We have noted that in the revised manuscript. The authors emphasize that application of physical constraints need not be dependent on a particular formulation of M.

   In terms of regularization, the revised manuscript also notes that

   - The elastostatic PDE solved in the manuscript encodes mass and momentum conservation (balance of internal forces) and the constitutive equations for linear elasticity in Eqn (1). The experimentally measured displacements are applied as boundary condition as explained in §2.3. The solution of this set of equations is expressed as the operator M. It is possible to directly invert the matrix M if the data lacks high frequency content (noise), giving a regularization-free solution (e.g. [3]). In practice, implicit (data interpolation, Fourier frequency cut-off) or explicit filtering (data smoothing, pre-processing, regularization) is almost always applied regardless of a particular TFM formulation, including FEM. Therefore, TFM method without regularization is not exclusive to FEM analysis.
   - Comparison of FEM methods with regularization and with other methods such as FTTC have been studied in detail [1, 4]. In summary, it was observerd that traction force reconstruction with Tikhonov-style regularization produced the least traction error with synthetic data. However, with experimental data, the issue noted by many [1, 4, 5] is the subjective choice of regularization parameter usually done with L-curve. This is precisely the topic of the manuscript to incorporate the regularization (hyper-) parameters as random variables in Bayesian framework to avoid explicit user-supplied value. Therefore, the authors focus on comparison with L-curve and that it doesnot exclude a particular framework such as FEM.
   - Finally, physical constraints such as conservation equations on a subset of domain (e.g. cell boundaries, force and moment balance) can be readily applied in the manuscript framework in the form of L matrix. The authors note that a constraint-based FEM formulation [6] such as

     $$\min\left(\frac{1}{2}||u-u^*||_2^2\right) \text{s.t}\Theta = 0$$

     is equivalent to a Lagrange multiplier approach,

     $$\min\left(\frac{1}{2}||u-u^*||_2^2 + \Theta\cdot\eta\right).$$

While a Lagrange multiplier approach enforces the constraint $\Theta = 0$ as a hard constraint. In case of an erroneous displacement value, this would lead to overfitting. This is ideal for high quality data or data that is pre-processed to avoid such high frequency content, but this pre-processing masks the underlying data confidence. In contrast, TFM-UQ approach would parameterize Θ constraint as a L operator and importantly, provide posterior uncertainty maps illustrating whether such constraint was applied more or less in certain regions [7].

The authors acknowledge that FEM comes with advantages such as applicability to complex geometries, ease of using non-linear and viscoelastic constitutive models. However, the follwing issues arise

- FEM methods not utilizing Tikhonov-style regularization often use explicit correction and regularization to displacement recovery step to reduce spurious noise [8]. Other work has implicit criteria to penalize 'outlier' forces that assigns lower penalty to higher forces (defined as 1.345 times the median of reconstructed force [7]). These approaches have thresholds or weight coefficients that affect regularization, which TFM users may or may not be able to control.
- FEM approaches use simple basis functions to interpolate variables between nodal points and to compute spatial derivatives and integrals. This approximation introduces implicit regularization influenced by the governing equations, nodal point resolution, chosen basis functions (e.g., linear vs. quadratic), and deformation gradient steepness. This implicit smoothing can be analyzed through spectral examination of the FEM operator, comparing the eigenvalues of the discrete operator M with those of the continuous differential operator. However, such analyses are typically semi-qualitative and not inherently included in FEM solvers.

In summary, discretizing the elastostatic equation by the FEM introduces different levels of explicit and implicit regularization. Explicit regularization such as Tikhonov removes the need for ad-hoc data pre-processing such as smoothing. Such regularization in any formulation can be indeed difficult to control and is often overlooked by TFM users, which is where presented Bayesian TFM method overcomes the ambiguity in regularization parameter selection.

5. Figures 5 and 6 show synthetic images of fluorescent particles in a substrate that are used for testing of the method. From the images, it appears that the densities of fluorescent particles are rather sparse. It is well known in image correlation methods like PIV that a denser distribution of speckles will create better measurements with higher spatial resolution and/or lower noise. Can the authors repeat the procedure using images that have a denser distribution of particles and compare the results using both their proposed method and other common methods for TFM?

   Authors' response: We agree with the reviewer's comment that higher density (upto saturation and clumping) of beads lead to higher fidelity in PIV measurements. That is precisely the motive to introduce a horizontal gradient in density as a way of modulating PIV noise in Fig 6. The revised manuscript includes a new Fig SI3 that applies the same analysis (Fig 6,7) to spatially uniform bead densities corresponding to the maximum and minimum density of the gradient in Fig 6. Briefly, TFM-UQ is qualitatively similar to traditional regularization with high density (low noise) spatially uniform images, however performs better in the case of low density (high noise) images by utilizing the PIV-UQ uncertainty information.

6. The first paragraph of p. 27 describes how the fluid mechanics community has developed the method referred to as PIV. The authors should mention the existence of other communities – the solid mechanics community uses the term "digital image correlation" and the computer vision community uses the term "optical flow." Details of the implementation of PIV, DIC, and optical flow can vary, but these different communities all have the same goal of obtaining kinematic data from images, so it is important to acknowledge that all communities have contributed.

   Authors' response: This point is well taken. The revised text now acknowledges the important contributions of those two communities, providing key references, and emphasizing hat TFM-UQ is agnostic to the specific method employed to measure deformation.

Reviewer #2

The authors present the first traction force microscopy (TFM) study that explicitly addresses the local uncertainty in the traction stress reconstruction. In line with similar efforts in the data sciences, they call their method "uncertainty aware TFM" and use the abbreviation "TFM-UQ" (for uncertainty quantification). In principle earlier TFM-methods based on Bayesian statistics (e.g. Refs. 9 or 21) already contained such a possibility, but it was never investigated before in the same depth as done here. Thus, this work is truly novel and fills an important gap in the TFM-field. The manuscript is well written, albeit very technical. The motivation for this work is nicely laid out and then the new methods are described, first the uncertainty treatment for

PIV (using a bootstrap method) and then for TFM itself (using a hierarchical Bayesian scheme). Both methods are validated first with simulated data and then with experimental cell data. Here micropatterning and the image correction of the Zeiss microscope are being used to check the disentangle the effect of imaging and TFM. It is shown that poor PIV results for the raw image, and that poor TFM-reconstruction results at low traction. As far as I can see, all these results are consistent and convincing.

Overall, this work is a nice advance in the TFM-field and I recommend to proceed towards publication. However, there are a few issues one should address before making a final recommendation.

Authors' response: We appreciate this reviewer's encouraging words and thoughtful suggestions. We have revised our manuscript incorporating all the reviewer's suggestions, as detailed below.

Major comments

1. This manuscript is rather technical in nature and would also fit to a journal on engineering, applied math or data sciences, at least in regard to the methods part. To improve the fit to PLOS CB, I suggest two measures. First, I note that on page 13, the computing time is recorded, but the authors should also explain in which language their code is written (Matlab, Cpp, Python ?) and which libraries are being used. Also, the code is not provided yet. This makes it difficult to assess how well this code can be used by the general reader. I suggest to include more comments on the code and how to use it, to make it a useful resource for readers who want to apply these advances.

   Authors' response: We appreciate the reviewer's comment on the accessibility of the code. Details about the code have been added in the revised manuscript. Moreover, a code repository has been made public before publication at https://github.com/adithank/TFM-UQ welcoming adoption by general users and contributions by TFM developers.

2. Second, I think that the life science community might want to learn more about the sources of uncertainty from the biological side. I encourage the authors to consider to include some experiments on different cell lines or inhibition experiments, and to show under which biological conditions uncertainty arises. Because the authors focus on the technical aspects, the biological aspects could be strengthened.

   Authors' response: This point is well taken. The revised manuscript now contains new experimental data of micropatterened fibroblast cells in different conditions (varying size of fibronectin islands), allowing readers to assess how TFM-UQ can be used to differentiate between biological variability and measurement uncertainty. The data is included as Fig SI.6 and is described in §3.3. Briefly, we see an increase in max. traction stress of fibroblast cells seeded on $75\mu m$ compared to $50\mu m$ at the population level (Fig SI.6 C). However, the uncertainty (quantified by posterior traction standard deviation) also increases for the $75\mu m$ island. Some datapoints ("outliers") on both sizes show larger errorbars at higher traction, but there is no observed correlation between pointwise traction magnitude and standard deviation (Fig SI.6 D). This data highlights that biological heterogeneity of cell-to-cell traction stresses is higher in the larger $75\mu m$ than the $50\mu m$ islands.

3. It is a well-known riddle in the field that DNA-sensor based measurements of traction forces give much more heterogeneous results, with some bonds experiencing exceedingly large forces (like 50 pN versus 5 pN baseline). However, it is not clear if these are artifacts of the method or real results. Does TFM-UQ give any hints to such large local forces? I suspect that the Gaussian assumption excludes statements on extreme values. Please explain.

   Authors' response: We agree with the reviewer that Gaussian priors used in TFM-UQ have narrow tails that severely penalize significant deviations from expected values. In the manuscript, by applying a hierarchical construction of the Gaussian prior whose precision parameter (α) is expressed as a gamma hyper-prior, the marginal prior distribution is effectively a student-t distribution (d.o.f = 2 for chosen value of flat hyperprior with $\theta = 1$). Therefore, while the hierarchical formulation allows relatively higher smoothness deviation compared to a strict Gaussian, the absolute value of such peak traction is decided by the global norm. This is a known limitation of one-parameter (global) shrinkage priors such as student-t or Lasso (equivalent to a Laplacian prior) especially in high-dimensional setting such as TFM [9]. An alternate prior selection is adaptive shrinkage or variable selection to tune a hyper-parameter to individual vectors that locally penalizes traction forces.

   While these heavy-tailed priors lead to non-conjugate posteriors making analytical solutions intractable, it should be relatively straightforward to extend TFM-UQ to handle this situation because the current β posterior is non-conjugate. Thus, TFM-UQ already implements an iterative Markov Chain Monte Carlo method to handle this type of sampling. In comparison to DNA haripin sensors such as [10] that can report forces due to single molecule adhesion, the authors speculate the resolution in nanometers and concentration of forces to a single ligand give rise to such heterogeneity. TFM force magnitude and localization improves with resolution (Fig 8, SI.4 and [11]). It is an interesting question whether such Gaussian prior is limiting when applied to displacement data in similar resolution (e.g. use of DNA

Fluorocube [12]) with high resolution microscopy and whether a locally adaptive shrinkage prior is required for that case. A different question is whether the substrate still behaves as a continuum at the very small lengthscales involved in these type of measurements, which come close to the pore size of polyacrylamide gels.

4. The authors assume "displacement noise", compare Eq. 2. They differ between two sources for noise, the uncertainty of the image processing which they can infer from the PIV and which might result from e.g. aggregates of fiducial beads; and other sources, like the elastostatic model. Is it clear that imaging really leads to additive noise in the displacement, for example if one could simulate the complete process of image generation? Similar for the mechanical noise: if the material law was not correct or if there were local heterogeneities in the polymer gel, would that also lead to an additive noise term in the displacement? In addition, I wonder if the two noises might be connected, like material heterogeneities leading to both imaging and mechanical noise? It would be interesting to learn if the authors have some thoughts on this set of questions.

   Authors' response: We appreciate the reviewer's insightful question on the modeling assumption of additive noise and uncertainty sources not related to imaging. We must concede that we do not have a definite answer to this question, but we speculate that heterogeneity in substrate material properties is a multiplicative error, since these properties enter M in equation (2). If such heterogeneities were associated with changes in, e.g., bead density, then there could be correlation between mechanical errors and imaging errors. It would be challenging to fully resolve these correlations, which depend on the imaging modality, optical settings, camera, substrate composition, etc, and are likely to vary widely among experiments. Instead, we adopt a bootstrap method to empirically determine the local PIV uncertainty after the image is formed. We believe this part of the model is quite robust and able to detect noisy areas caused by, e.g., bead clumps (Figure 9).

   Performing Bayesian inference with multiplicative noise in Young's modulus, Poisson's ratio, substrate thickness, etc is challenging. First, handling multiplicative noise in Bayesian inference is significantly more complex than additive noise. Second, it requires modeling uncertainty in all these mechanical parameters. While a useful step toward applicability of a fully Bayesian TFM model, the state-of-art global additive noise model (ε_β) is less ideal than PIV uncertainty treatment, as it does not capture spatial variations. Working towards incorporating multiplicative noise, incorporating nonzero covariance (off-diagonal terms) in ε_β could an effective compromise solution worth future investigation. The non-zero covariance would account for the spatial propagation of the effects of, e.g., substrate stiffness heterogeneities. The hyperparameters of this multivariate Gaussian could be empirically tuned using correlations in the experimental data and considered fixed during inference, a common approach in hierarchical Bayesian models [13]. Some of these ideas are now included in the revised discussion.

Minor comments

1. Fig. 5: it is not clear to me how large the ensembles are and if the TFM-UQ also averages over the same ensembles? For a TFM-experiment, one has only one shot and it was my understanding that TFM-UQ uses only one image pair for bootstrapping. Please clarify.

   Authors' response: We appreciate the reviewer highlighting about the one-shot nature of experimental TFM data, which is the motivation for bootstrap method. However, for validation of PIV-UQ in Fig 5, the authors generate an ensemble of synthetic images of a fixed displacement field where the only variability stems from the random distribution of beads and a random image pixel noise. TFM-UQ $\sigma_{u,PIV}$ estimate in Fig 5 is the RMS of $\sigma_{u,PIV}$ of all such realizations. This process is explained in detail in Fig SI 1. The revised manuscript emphasizes this point in §3.1.

   The synthetic validation had an ensemble of 50 1024×1024 images. PIV analysis with a window size of 32x32 results in 198,450 datapoints and a 64x64 results in 48,050 datapoints (Fig 5E). This is clarified in main text and Fig SI 1.

   Fig. 6C: coloring and legend are not clear, should this not be a horizontal gradient?

   Authors' response: The legend (right triangle) denoting the level of noise simulated is rotated to align with the gradient direction of simulated noise as suggested by the reviewer for clarity.

Data accessibility

Have the authors made all data and (if applicable) computational code underlying the findings in their manuscript fully available?

The PLOS Data policy requires authors to make all data and code underlying the findings described in their manuscript fully available without restriction, with rare exception (please refer to the Data Availability Statement in the manuscript PDF

file). The data and code should be provided as part of the manuscript or its supporting information, or deposited to a public repository. For example, in addition to summary statistics, the data points behind means, medians and variance measures should be available. If there are restrictions on publicly sharing data or code —e.g. participant privacy or use of data from a third party—those must be specified.

Reviewer #1: Yes

Reviewer #2: No: The Github link does not work yet and the code is not described, see my report.

References

[1] Kulkarni AH, Ghosh P, Seetharaman A, Kondaiah P, Gundiah N. Traction cytometry: regularization in the Fourier approach and comparisons with finite element method. Soft matter. 2018;14(23):4687–4695.

[2] Kybic J. Bootstrap resampling for image registration uncertainty estimation without ground truth. IEEE Transactions on Image Processing. 2010;19:64–73. doi:10.1109/tip.2009.2030955.

[3] Butler JP, Toli-Nørrelykke IM, Fabry B, Fredberg JJ. Traction fields, moments, and strain energy that cells exert on their surroundings. American Journal of Physiology - Cell Physiology. 2002;282:595–605. doi:10.1152/ajpcell.00270.2001.

[4] Apolinar-Fernández A, Blázquez-Carmona P, Ruiz-Mateos R, Barrasa-Fano J, Van Oosterwyck H, Reina-Romo E, et al. Regularization techniques and inverse approaches in 3D Traction Force Microscopy. International Journal of Mechanical Sciences. 2024;283:109592. doi:10.1016/j.ijmecsci.2024.109592.

[5] Sabass B, Gardel ML, Waterman CM, Schwarz US. High Resolution Traction Force Microscopy Based on Experimental and Computational Advances. Biophysical Journal. 2008;94:207–220. doi:10.1529/biophysj.107.113670.

[6] Sanz-Herrera JA, Barrasa-Fano J, Cóndor M, Van Oosterwyck H. Inverse method based on 3D nonlinear physically constrained minimisation in the framework of traction force microscopy. Soft Matter. 2021;17(45):10210–10222. doi:10.1039/D0SM00789G.

[7] Steinwachs J, Metzner C, Skodzek K, Lang N, Thievessen I, Mark C, et al. Three-dimensional force microscopy of cells in biopolymer networks. Nature Methods 2015 13:2. 2015;13:171–176. doi:10.1038/nmeth.3685.

[8] Legant WR, Choi CK, Miller JS, Shao L, Gao L, Betzig E, et al. Multidimensional traction force microscopy reveals out-of-plane rotational moments about focal adhesions. Proceedings of the National Academy of Sciences. 2013;110(3):881–886. doi:10.1073/pnas.1207997110.

[9] Yi N, Ma S. Hierarchical shrinkage priors and model fitting for high-dimensional generalized linear models. Statistical applications in genetics and molecular biology. 2012;11(6). doi:10.1515/1544-6115.1803.

[10] Blakely BL, Dumelin CE, Trappmann B, McGregor LM, Choi CK, Anthony PC, et al. A DNA-based molecular probe for optically reporting cellular traction forces. Nature Methods 2014 11:12. 2014;11:1229–1232. doi:10.1038/nmeth.3145.

[11] Mollica MY, Beussman KM, Kandasamy A, Rodríguez LM, Morales FR, Chen J, et al. Distinct platelet F-actin patterns and traction forces on von Willebrand factor versus fibrinogen. Biophysical Journal. 2023;122:3738–3748. doi:10.1016/j.bpj.2023.07.006.

[12] Mortazavi A, Jiang J, Laric P, Helmerich DA, Seifert R, Sauer M, et al. Traction Force Microscopy with DNA FluoroCubes. bioRxiv. 2024;doi:10.1101/2024.04.12.589182.

[13] Banerjee S, Carlin BP, Gelfand AE. Hierarchical modeling and analysis for spatial data. Chapman and Hall/CRC; 2003.
